# Supplementary material for: Comparative genomics provides new insights into the diversity, physiology, and sexuality of the only industrially exploited tremellomycete: Phaffia rhodozyma
Source: BMC Genomics. 2016 Nov 9;17:901. doi: 10.1186/s12864-016-3244-7 (PMC5103461; doi:10.1186/s12864-016-3244-7)
Supplement: Additional file 6: — List of orphan genes with links to PFAM (related to Additional file 1: Table S1). (ZIP 1428 kb) [file 12864_2016_3244_MOESM6_ESM.zip › PFAM_UNIQUE/G05272_P.html]

#                                                                            --- full sequence --- -------------- this domain -------------   hmm coord   ali coord   env coord
  
# target name        accession   tlen query name           accession   qlen   E-value  score  bias   #  of  c-Evalue  i-Evalue  score  bias  from    to  from    to  from    to  acc description of target
  
#------------------- ---------- ----- -------------------- ---------- ----- --------- ------ ----- --- --- --------- --------- ------ ----- ----- ----- ----- ----- ----- ----- ---- ---------------------
  
G05272\_P             -            143 Gly-zipper\_OmpA      PF13436.1    118     0.028   12.8   1.9   1   2     0.065        33    2.9   0.0    44    69    49    74    26    79 0.67 - Glycine-zipper containing OmpA-like membrane domain  
G05272\_P             -            143 Gly-zipper\_OmpA      PF13436.1    118     0.028   12.8   1.9   2   2   0.00098      0.49    8.7   1.1    65    99    97   130    90   140 0.74 - Glycine-zipper containing OmpA-like membrane domain
